# The Transcriptional Repressor BS69 is a Conserved Target of the E1A Proteins from Several Human Adenovirus Species

**DOI:** 10.3390/v10120662

**Published:** 2018-11-22

**Authors:** Ali Zhang, Tanner M. Tessier, Kristianne J. C. Galpin, Cason R. King, Steven F. Gameiro, Wyatt W. Anderson, Ahmed F. Yousef, Wen T. Qin, Shawn S. C. Li, Joe S. Mymryk

**Affiliations:** 1 Department of Microbiology and Immunology, The University of Western Ontario, London, ON N6A 3K7, Canada; azhang44@uwo.ca (A.Z.); ttessie2@uwo.ca (T.M.T.); kgalpin@ohri.ca (K.J.C.G.); cking46@uwo.ca (C.R.K.); sgameiro@uwo.ca (S.F.G.); wander@uwo.ca (W.W.A.); 2 Department of Chemistry, Khalifa University, 54224 Abu Dhabi, UAE; ahmed.yousef@ku.ac.ae; 3 Department of Biochemistry, The University of Western Ontario, London, ON N6A 3K7, Canada; wqin24@uwo.ca (W.T.Q.); sli@uwo.ca (S.S.C.L.); 4 Department of Oncology, The University of Western Ontario, London, ON N6A 3K7, Canada; 5 London Regional Cancer Program, Lawson Health Research Institute, London, ON N6C 2R5, Canada

**Keywords:** human adenovirus, E1A, BS69, ZMYND11, short linear motifs, transcriptional regulation

## Abstract

Early region 1A (E1A) is the first viral protein produced upon human adenovirus (HAdV) infection. This multifunctional protein transcriptionally activates other HAdV early genes and reprograms gene expression in host cells to support productive infection. E1A functions by interacting with key cellular regulatory proteins through short linear motifs (SLiMs). In this study, the molecular determinants of interaction between E1A and BS69, a cellular repressor that negatively regulates E1A transactivation, were systematically defined by mutagenesis experiments. We found that a minimal sequence comprised of MPNLVPEV, which contains a conserved PXLXP motif and spans residues 112–119 in HAdV-C5 E1A, was necessary and sufficient in binding to the myeloid, Nervy, and DEAF-1 (MYND) domain of BS69. Our study also identified residues P113 and L115 as critical for this interaction. Furthermore, the HAdV-C5 and -A12 E1A proteins from species C and A bound BS69, but those of HAdV-B3, -E4, -D9, -F40, and -G52 from species B, E, D, F, and G, respectively, did not. In addition, BS69 functioned as a repressor of E1A-mediated transactivation, but only for HAdV-C5 and HAdV-A12 E1A. Thus, the PXLXP motif present in a subset of HAdV E1A proteins confers interaction with BS69, which serves as a negative regulator of E1A mediated transcriptional activation.

## 1. Introduction

Protein products of the early region 1A (E1A) gene are the first viral proteins produced upon infection by human adenoviruses (HAdV) [1]. Although the largest E1A isoform is smaller than 300 amino acids, it directly binds over 30 host factors [2,3]. Using these interactions, E1A dysregulates cell cycle control, protein localization, and gene expression within the host cell [4,5,6,7,8,9]. E1A is divided into four conserved regions (CR), termed CR1 through CR4 [10]. In HAdV-C5, most of E1A is intrinsically disordered, with the exception of a zinc finger in CR3 and an alpha helical motif close to the N-terminus [2]. The zinc finger in CR3 is especially important in the ability of E1A to activate transcription because it facilitates the formation of the transcription pre-initiation complex at target promoters and stimulates transcriptional elongation [11,12]. The disordered regions of E1A are comprised of an abundance of short linear motifs (SLiMs). SLiMs are typically fewer than 10 residues in length, but can flexibly mediate a wide variety of interactions with other protein targets [13,14,15,16]. Despite its compact size of only 23 amino acids, CR2 of E1A is extremely dense with SLiMs. This region contains the LXCXE and EVIDLT motifs used to bind the retinoblastoma protein (Rb) [17,18,19] and SUMO conjugase UBC9 [20], respectively. The LXCXE motif has also been recently reported to mediate an interaction between E1A and the stimulator of interferon genes (STING) [21]. The final known SLiM within CR2 is the PXLXP motif, which is used to bind the transcriptional repressor BS69 [22,23]. Importantly, these motifs are either in close proximity, or actually overlapping in the primary sequence of HAdV-C5 E1A [24]. The density of these interaction motifs suggests that the utilization of SLiMs is a useful strategy for maximizing the functionality of limited viral coding capacity.

BS69 was initially discovered as a HAdV-C5 E1A interacting protein and reported to function as a strong inhibitor of E1A-dependent transactivation [22]. This 69 kDa protein is 602 residues in length, localizes to the nucleus, is ubiquitously expressed, and carries out a variety of functions associated with gene regulation [25,26,27,28]. BS69 is composed of four previously described domains. The plant homeodomain (PHD), bromodomain (BROMO), and PWWP (named after the conserved Pro-Trp-Trp-Pro motif) domains are involved in binding to various post-translationally modified histone tails, while the myeloid, Nervy, and DEAF-1 (MYND) domain is a zinc finger that mediates protein-protein interactions [29,30]. Although the MYND domain by itself is sufficient for interaction with E1A, it is not known if the other domains are required for BS69 to repress E1A transactivation.

In addition to E1A, BS69 binds to several other PXLXP-containing proteins including members of the zinc-fingers and homeoboxes (ZHX) family and m-Bop, although the involvement of the PXLXP motif in these interactions has not been confirmed [31,32,33]. Another example of BS69-binding proteins include ETS1 and ETS2, which are cellular signal-dependent transcription factors that regulate transcription downstream of the Ras/MAPK pathway [34,35]. In this case, BS69 binds to and inhibits transactivation by ETS2, but does not bind nor affect ETS1 dependent activation [36]. Furthermore, EBNA2, a transcriptional activator encoded by Epstein-Barr virus (EBV), binds BS69 via two PXLXP motifs and is similarly repressed by BS69 [25]. Data from those studies indicate that although the core PXLXP motif is necessary in mediating an interaction with BS69, additional residues in and around this core motif also appear crucial for interaction.

Despite the original identification of the interaction between E1A and BS69 in 1995 [22], the interaction surface and mechanism by which BS69 inhibits E1A-mediated transactivation remains only partially characterized. In this study, we set out to map this binding surface in more detail, and identified residues 112–119 in HAdV-C5 E1A as the minimal BS69 interaction motif. Notably, all previous studies on the E1A-BS69 interaction have been performed only using HAdV-C5 E1A [22,23,37,38,39]. As such, we set out to determine if the interaction with BS69 is conserved in the E1A proteins from other HAdV species. We report here that the E1A proteins of HAdV-C5 and -A12 (from HAdV species C and A) bound BS69, but those of HAdV-B3, -E4, -D9, -F40, and -G52 (from species B, E, D, F, and G respectively) did not. Interestingly, E1A proteins from several species A HAdVs contain multiple BS69 interaction motifs. Functionally, BS69 repressed E1A-mediated transactivation, but only for those E1A proteins to which it binds. Thus, BS69 functions as a negative regulator of E1A-mediated activation in a subset of HAdV species. Taken together, our study expands upon the molecular characterization of this SLiM, provides insight into its biological role in a viral context and enhances our understanding of the evolution of SLiMs as protein interaction surfaces in general.

## 2. Materials and Methods

### 2.1. Plasmid Constructs

CR2 of HAdV-B3 (residues 85–150), -E4 (residues 91–145), -C5 (residues 93–139), -D9 (residues 84–138), -A12 (residues 80–125), -F40 (residues 78–132), and -G52 (residues 78–130) were cloned using PCR to amplify the specified region from full-length E1A and ligated into the pBAIT backbone vector [40]. The various E1A fragments and substitution mutants were generated using self-annealing oligonucleotides that were inserted into the pBAIT backbone vector to generate LexA DNA-binding domain (DBD) fusion proteins. These short E1A fragments were designed with a preceding flexible “SGG” linker to minimize the possibility of steric interference from the DBD. Alanine scanning mutants were constructed using overlap extension site-directed mutagenesis [41] and cloned into the pBAIT backbone vector. The wildtype (WT) BS69 construct (residues 46–602) was kindly provided by Dr. Rene Bernards (Netherlands Cancer Institute, Amsterdam, The Netherlands) and the MYND domain (residues 427–602) was amplified using PCR and cloned into the *EcoR*I and *Sal*I sites of pJG4-5 (Clontech, Palo Alto, CA, USA) to fuse BS69 to a B42 activation domain. The pSH18-34 (Invitrogen, Carlsbad, CA, USA) reporter plasmid contains 8× LexA binding sites upstream of a LacZ reporter gene. Sequences encoding the largest E1A isoform of HAdV-C5, -D9, -A12 and -F40, as well as L115 HAdV-C5 E1A were cloned into the pM (Clontech, Palo Alto, CA, USA) backbone vector to generate GAL4 DBD (residues 1–147) fusion proteins. BS69 wildtype (residues 46–602), MYND (residues 427–602), and ∆MYND (residues 46–426) constructs were cloned using PCR to amplify the specified region and ligated into a modified pcDNA3 vector (Invitrogen by Thermo Fisher Scientific, Carlsbad, CA, USA) that includes an N-terminal HA tag. The pGL2-(GAL4)_6_-Luc reporter vector consists of 6× GAL4 binding sites and a minimal TATA box upstream of a luciferase reporter gene. The largest E1A isoforms of HAdV-B3, -E4, -C5, -D9, -A12, -F40, and -G52 were cloned into a pEGFP backbone vector to generate E1A proteins with an N-terminal GFP tag as previously described [9].

### 2.2. Yeast Two-Hybrid Assays

Yeast culture and transformation were completed as previously described [42]. W303-1A (*MATa leu2-3,112 trp1-1 can1-100 ura3-1 ade2-1 his3-11,15*) yeast used for yeast two-hybrid experiments were transformed using the lithium acetate protocol as previously described [43]. These assays were carried out using yeast transformed with equal concentrations of pBAIT constructs, pJG4-5 constructs, and the reporter plasmid pSH18-34. Transformed yeast were grown overnight in 30 °C using galactose as the carbon source to induce expression of the GAL1 driven PJG4-5 vector. To measure protein-protein interactions, yeast two-hybrid assays were completed as previously described using ortho-Nitrophenyl-β-galactoside (ONPG) as the substrate [42]. Results are shown in absolute Miller units or as percent binding compared to a positive control.

### 2.3. Western Blots

Yeast protein extractions were prepared as previously described [44]. The samples were then combined with yeast loading buffer (0.25 M Tris-HCl pH 6.8, 50% glycerol, and 0.05% bromophenol blue) before being analyzed by Western blot. HT1080 cell lysates were collected using NP-40 lysis buffer (0.5% NP-40, 150 mM NaCl, 50 mM Tris-HCl, pH 7) supplemented with 0.5% protease inhibitor cocktail (Sigma-Aldrich, St. Louis, MO, USA). Protein concentrations were determined using Protein Assay Dye Reagent (Bio-Rad Laboratories, Hercules, CA, USA) using BSA as standards.

Protein samples were resolved using NuPAGE 4–12% Bis-Tris polyacrylamide gradient gels (Invitrogen by Thermo Fisher Scientific, Carlsbad, CA, USA) and subsequently transferred to a polyvinylidene fluoride (PVDF) membrane (GE Healthcare, Chicago, IL, USA) for protein detection. Membranes were blocked at room temperature for one hour using 5% skim milk in TBS-T (20 mM Tris, 136 mM NaCl, and 0.1% Tween-20) and incubated overnight at 4 °C with primary antibody in 5% skim milk in TBS-T. Subsequently, the membranes were incubated at room temperature for 30 min with secondary antibody in 5% skim milk in TBS-T. Proteins were detected using Luminata Crescendo Western HRP Substrate (Merck Millipore, Burlington, MA, USA). Images were developed on Amersham Hyperfilm (GE Healthcare, Chicago, IL, USA) using an automated film processor (Konica Minolta SRX-101A) according to the manufacturer’s protocol.

For the yeast two-hybrid assays, LexA-E1A bait proteins were detected with rabbit anti-LexA (1:10,000, Millipore 06-719), BS69-B42 prey proteins were detected using rat anti-HA (1:2000, Roche 11867423001), and rabbit anti-glucose-6-phosphate dehydrogenase (G6PD) (1:100,000, Sigma A-9521) was used as the loading control. For coimmunoprecipitation (CoIP) assays, GFP-E1A proteins were detected using rabbit anti-GFP (1:2000, Clontech 632592), and BS69-HA proteins were detected using rat anti-HA (1:2000, Roche 11867423001). Goat anti-rabbit IgG (1:10,000, Jackson Laboratories 111-035-003) and goat anti-rat IgG (1:200,000, Pierce 31470) secondary antibodies conjugated to horseradish peroxidase were used to visualize the protein bands.

### 2.4. Cell Culture

HT1080 fibrosarcoma cells (ATCC # CCL-121) were cultured in Dulbecco’s modified eagle medium supplemented with 10% fetal bovine serum, 100 IU/mL penicillin, and 100 μg/mL streptomycin in a 37 °C incubator with 5% CO2.

### 2.5. Coimmunoprecipitation

HT1080 cells were cultured as above and transfected with vectors expressing GFP-tagged full-length E1A and HA-tagged BS69 MYND domain. Transfections were carried out using X-tremeGene HP DNA Transfection Reagent (Roche, Basel, SUI) using a 1:2 DNA to transfection reagent ratio according to the manufacturer’s protocol. A total of 2 μg of each plasmid was used except for HAdV-G52, for which only 1 μg was used to ensure similar levels of protein expression between transfections. Cells were harvested 24 h post-transfection and lysed using NP-40 lysis buffer supplemented with 0.5% Protease Inhibitor Cocktail (Sigma-Aldrich, St. Louis, MO, USA). Cell lysates were incubated with 1 μL of rabbit anti-GFP antibody (632592, Clontech, Palo Alto, CA, USA) and 120 µL of a 10% Sepharose-Protein A slurry (Sigma-Aldrich, St. Louis, MO, USA) overnight at 4 °C with nutating. Samples were then washed five times with NP-40 lysis buffer, resuspended in 1× NuPAGE LDS Sample Buffer (Thermo Fisher Scientific, Carlsbad, CA, USA), and boiled for 5 min before being analyzed using Western blotting.

### 2.6. Luciferase Assays

For luciferase assays, 3 × 10^5^ HT1080 cells were seeded per well on 6-well plates. Cells were transfected 24 h after seeding with 0.05 μg of GAL4-E1A construct in the pM backbone, increasing concentrations of BS69-HA construct in the pcDNA3-HA backbone, 0.5 μg of pGL2-(GAL4)_6_-Luc reporter vector, and empty pcDNA3-HA vector as required to ensure a total amount of 2 μg DNA transfected per well. Cells were harvested 24 h post-transfection and lysed using Luciferase Cell Culture Lysis Reagent (Promega, Madison, WI, USA). Relative luciferase units were detected using Luciferase Assay Substrate (Promega, Madison, WI, USA) with the Lumat LB 9507 luminometer (Berthold Technologies, Bad Wildbad, Germany) with measuring time set to 10 s. Luciferase activity was normalized to total protein concentration, determined by using Protein Assay Dye Reagent (Bio-Rad Laboratories, Hercules, CA, USA) with BSA as standards, then calculated as fold increase over that of cells transfected with an empty pM vector.

### 2.7. Statistical Analysis

**Figure 1:**
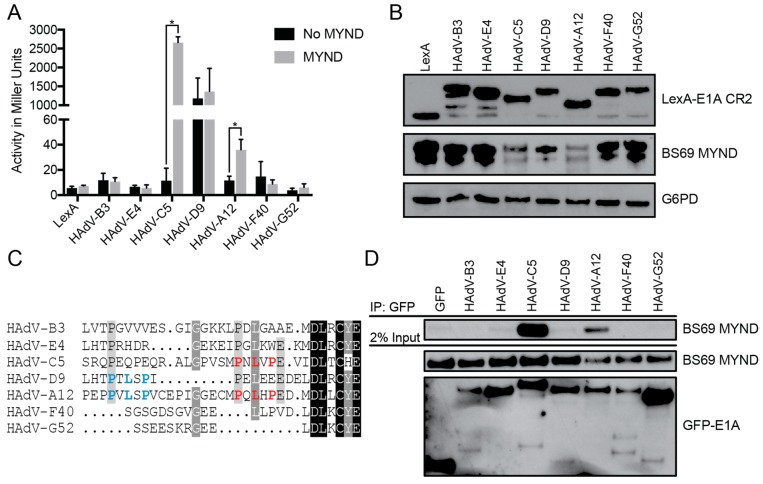
The interaction between early region 1A (E1A) and BS69 is present in only a subset of human adenovirus (HAdV) species. (**A**) Yeast two-hybrid analysis of the ability of the E1A CR2 portions from representative HAdV types to bind BS69. Results are shown as mean ± SD, *n* = 3. Significance markers are assigned in comparison to the no myeloid, Nervy, and DEAF-1 (MYND) control for each respective E1A type (* *p* ≤ 0.05). (**B**) Western blot detection of expression of the LexA-DBD E1A CR2 fusions and the BS69 MYND construct in yeast. G6PD was used as a loading control. (**C**) Multiple sequence alignment of E1A CR2 proximal regions of HAdV-B3, -E4, -C5, -D9, -A12, -F40, and -G52. Residues with high conservation are shaded in black, with darker shading indicating higher levels of conservation. The previously identified PXLXP motif contributing to HAdV-C5 E1A interaction with BS69 and the corresponding motif in HAdV-A12 E1A are indicated in red. A second N-terminal PXLXP motif present in the E1A proteins of HAdV-D9 and -A12 is indicated in blue. (**D**) Coimmunoprecipitation analysis of the ability of E1A proteins from representative HAdV types to bind BS69 in mammalian cells. Vectors expressing GFP-tagged full-length E1A and HA-tagged BS69 MYND were co-transfected into human HT1080 cells. Cell lysates were subjected to immunoprecipitation using an anti-GFP antibody. Western blots were probed with anti-GFP and anti-HA antibodies.

For yeast two-hybrid assays using the CR2 regions of different HAdV E1As (Figure 1A), significance was calculated pairwise between each E1A fragment as bait with empty prey vector and the same E1A fragment as bait with BS69 prey vector using individual *T*-tests, with *p* ≤ 0.05 considered significant. For the remaining yeast two-hybrid assays, significance was calculated using one-way ANOVA with Dunnett’s multiple comparisons test, with *p* ≤ 0.05 considered significant. For BS69-mediated repression of transactivation significance was calculated by two-way ANOVA with Dunnett’s multiple comparisons test, with *p* ≤ 0.05 considered significant.

## 3. Results

### 3.1. The Interaction between E1A and BS69 is Conserved Amongst HAdV Species C and A

Previous studies on the interaction between E1A and BS69 were performed using only HAdV-C5 E1A [22,23,37,38,39]. As many of E1A’s interactions with cellular proteins are crucial for successful virus propagation, they are often conserved amongst other adenovirus types [3,45]. An example of this is the interaction between E1A and Rb mediated by E1A’s LXCXE SLiM [19]. Binding and subsequent deactivation of Rb by E1A is crucial for cell cycle deregulation during infection, and this SLiM is present in the E1A proteins of all known HAdVs [46,47,48,49,50,51]. Thus, we set out to determine if the interaction between BS69 and E1A was similarly conserved using E1As from HAdV-B3, -E4, -C5, -D9, -A12, -F40, and -G52, representing all seven HAdV species (B, E, C, D, A, F, and G respectively).

We used a series of yeast two-hybrid assays to determine the relative binding affinity between BS69 and the E1A proteins from the different HAdV types. The interaction between E1A and BS69 appears to be conserved in HAdV-C5 and -A12, but not HAdV-B3, -E4, -D9, -F40, and -G52 (Figure 1A). Unlike the CR2 regions of the other species, HAdV-D9 CR2 functioned as a strong activator of transcription when fused to the LexA-DBD in the absence of the BS69 prey vector. Thus, this E1A protein differs from the others in that it contains an intrinsic transcriptional activation domain that functions to recruit the RNA polymerase II transcriptional apparatus when tethered to DNA via the LexA-DBD in yeast. However, there was no significant increase in activity when co-expressed with the BS69 prey vector. All E1A fusions with the LexA-DBD were expressed at comparable levels (Figure 1B). Binding of BS69 strongly corresponds with the presence of a PXLXP motif in the alignment at a position corresponding to that found in HAdV-C5 E1A (indicated in red in the sequence alignment in Figure 1C) while species lacking this motif did not bind BS69. Inspection of the protein sequences revealed that HAdV-A12 E1A contains a second PXLXP motif 10 residues N-terminal to the one aligning with HAdV-C5 E1A (as indicated in blue in Figure 1C). Interestingly, this second motif in HAdV-A12 E1A CR2 also aligns with a PXLXP motif present in HAdV-D9 E1A CR2 (Figure 1C). However, this motif appears to be non-functional in HAdV-D9 E1A CR2 since this E1A protein fails to bind BS69 (Figure 1A).

To verify these results in a mammalian context, we tested the ability of full-length E1A from each representative HAdV type to bind with the MYND domain of BS69 using coimmunoprecipitation (CoIP) assays in extracts from cultured mammalian cells. Similar to the results from yeast two-hybrid tests performed using portions of E1A containing CR2, full-length HAdV-C5 and -A12 E1A bound to BS69 in these experiments. Despite the presence of a PXLXP motif in HAdV-D9, it did not interact with BS69 in this assay. Similarly, no interaction was observed with full-length HAdV-B3, -E4, -F40, and -G52 E1A and BS69 (Figure 1D). Collectively, these analyses confirm that E1A proteins from only a subset of HAdV types, specifically species A and C, target BS69. Furthermore, these results demonstrate that the simple presence of a PXLXP motif in CR2 is not sufficient to predict interaction with BS69.

### 3.2. Characterizing the Molecular Determinants Needed for HAdV-C5 E1A to Bind to BS69

**Figure 2:**
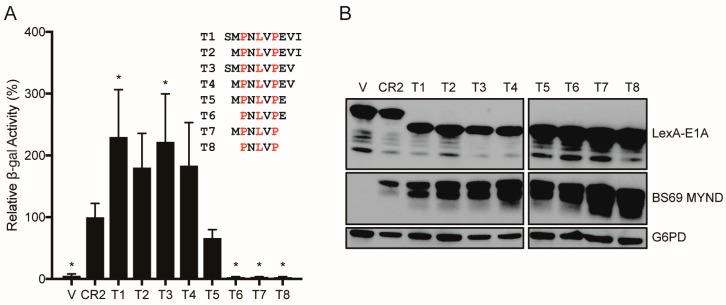
Mapping the minimal BS69 interacting region in HAdV-C5 E1A CR2. (**A**) Vectors expressing the indicated E1A truncation mutants and BS69 MYND domain were transformed as bait and prey respectively in yeast. Sequences of the E1A truncation mutants, T1–T8, are shown in the legend beside the graph. V = vector control, where HAdV-C5 E1A CR2 was co-transformed with an empty prey vector. Results are shown as mean ± SD of percent activity normalized to E1A CR2, *n* = 4. Significance markers are assigned in comparison to the CR2 positive control (* *p* ≤ 0.05). (**B**) Western blot of yeast cell lysates to confirm protein expression. Two blots were run concurrently in parallel to include all the samples. Bait and prey proteins were visualized using anti-LexA DBD and anti-HA antibodies respectively. G6PD was used as a loading control. V = vector control.

**Figure 3:**
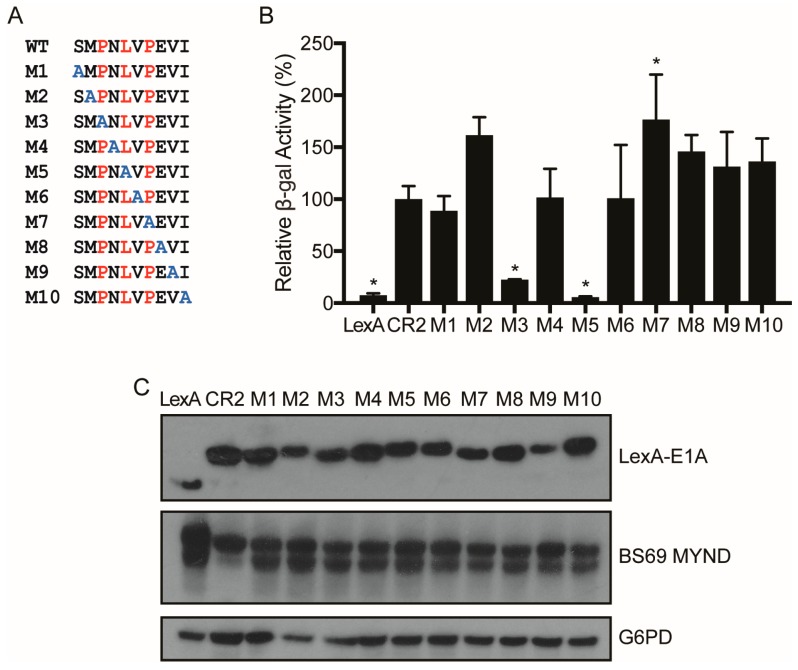
Determination of specific residues of HAdV-C5 E1A CR2 required to interact with BS69. (**A**) Sequences of the E1A CR2 point mutants, M1–M10, used to determine the importance of specific residues within and adjacent to the HAdV-C5 E1A PXLXP motif for interaction with BS69. (**B**) Vectors expressing E1A CR2 point mutants and BS69 MYND were transformed as bait and prey respectively in yeast. Results are shown as mean ± SD of percent activity normalized to HAdV-C5 CR2, *n* = 3. Significance markers are assigned in comparison to the CR2 positive control (* *p* ≤ 0.05). (**C**) Western blot of yeast cell lysates to confirm protein expression. Bait and prey proteins were visualized using anti-LexA DBD and anti-HA antibodies respectively. G6PD was used as a loading control.

As described above, E1A is a multifunctional protein that utilizes an arsenal of SLiMs to interact with and perturb the function of various target host factors. Previous studies have shown that the PXLXP motif within CR2 of HAdV-C5 E1A is involved in mediating the interaction with BS69 [23]. However, the exact molecular determinants of this interaction have not been defined. Thus, we set out to determine the minimal interacting region, as well as the specific residues used by HAdV-C5 E1A to interact with BS69. To determine the minimal interacting region, we tested a series of HAdV-C5 E1A short peptide truncation mutants denoted T1 through T8 ranging from residues 111–120 (sequence: SMPNLVPEVI) to residues 113–117 (sequence: PNLVP) for interaction with BS69 using yeast two-hybrid assays (Figure 2A). The T1 peptide bound BS69 at least as well as the full CR2 region in this assay. Peptides lacking the S111, I120 or both residues (T2, T3, and T4) retained strong binding activity with BS69. Further truncation by removal of the V119 residue (T5) greatly reduced binding. Truncation mutants T6–T8 had no detectable interaction with BS69. Western blot analysis confirmed that all constructs were expressed at similar levels (Figure 2B). Thus, additional residues adjacent to the core PXLXP motif are also required for interaction with BS69, and the minimal interacting region appears to be the 8-amino acid sequence MPNLVPEV. To determine the contribution of specific residues within this region for binding to BS69, we tested a series of alanine scanning mutants (Figure 3A). These mutants contain a single substitution of alanine at each of the 10 positions in the peptide, spanning residues 111–120 of HAdV-C5 E1A (M1–M10) in the context of CR2. Surprisingly, this analysis suggests that the BS69-binding SLiM is relatively tolerant to alanine substitutions since most mutants bound indistinguishably from the WT peptide. These results verified that the PXLXP motif is essential in mediating this interaction. Accordingly, the P113A (M3) and L115A (M5) mutants exhibited reduced interaction with BS69 by 4- and 18-fold compared to the WT control (Figure 3B). Surprisingly, the P117A (M7) mutant motif did not significantly decrease interaction, even though it is a part of the PXLXP core motif. All mutant proteins were expressed (Figure 3C). Collectively, this analysis confirms that P113 and L115 play key roles in the interaction with BS69, and reveals a surprising tolerance for alanine substitutions at other positions in this SLiM.

### 3.3. Characterizing the Molecular Determinants Needed for Species A HAdV E1A Interactions with BS69

**Figure 4:**
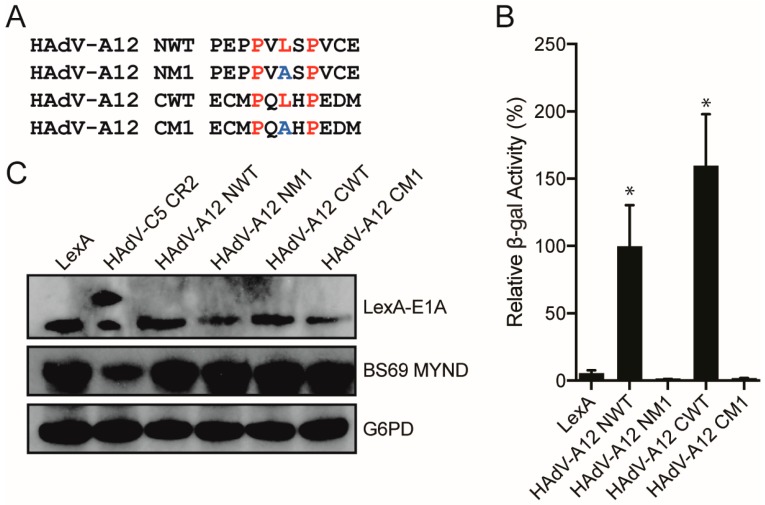
Analysis of BS69 interaction with the two putative PXLXP motifs present in the HAdV-A12 E1A protein. (**A**) Sequences of the motifs corresponding to the wildtype (WT) and mutant N-terminal and C-terminal PXLXP motifs in HAdV-A12 E1A tested. Putative PXLXP motifs are indicated in red and mutations in the motifs are indicated in blue. (**B**) Vectors expressing the motifs depicted in (A) and BS69 MYND were tested for interaction with BS69 by yeast two-hybrid analyses. Results are shown as mean ± SD of percent activity normalized to the HAdV-A12 N-terminal WT positive control, *n* = 3. Significance markers are assigned in comparison to the LexA negative control (* *p* ≤ 0.05). (**C**) Western blot of yeast cell lysates to confirm protein expression. Bait and prey proteins were visualized using anti-LexA DBD and anti-HA antibodies respectively. G6PD was used as a loading control.

Next, we sought to characterize the multiple PXLXP motifs in CR2 of HAdV-A12 E1A to determine if they each independently confer interaction with BS69. We created truncation mutants composed of the PXLXP core motif with three flanking residues for each of the two motifs within HAdV-A12 E1A (N-terminal residues: 80–90; C-terminal residues: 95–105), as well as a leucine to alanine point mutant within each respective core PXLXP motif (Figure 4A). Each HAdV-A12 E1A expression construct was expressed in yeast with the BS69 MYND domain and yeast two-hybrid tests were performed. The wildtype (WT) constructs for both the N-terminal and C-terminal HAdV-A12 E1A motifs bound to BS69, and the central leucine residue in these PXLXP motifs was necessary in each motif for interaction with BS69 (Figure 4B). All mutant proteins were expressed (Figure 4C). Thus, HAdV-A12 E1A contains two independent BS69 interaction motifs.

**Figure 5:**
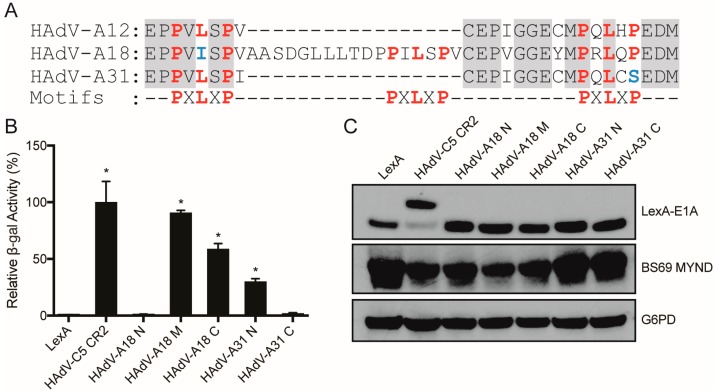
Analysis of BS69 interaction with the E1A proteins from HAdV-A18 and -A31. (**A**) Sequence alignment of the relevant portions of HAdV-A12, -A18, and -A31. PXLXP like sequences are indicated in red, with divergent residues indicated in blue. (**B**) The indicated fragments corresponding to the N-terminal (N), middle (M), and C-terminal (C) PXLXP motifs in HAdV-A18 and -A31 E1A were tested for interaction with BS69 MYND by yeast two-hybrid analyses. Results are shown as mean ± SD of percent activity normalized to HAdV-C5 CR2 positive control, *n* = 3. Significance markers are assigned in comparison to the LexA negative control (* *p* ≤ 0.05). (**C**) Western blot analysis of expression of the motifs tested.

We next tested fragments corresponding to the WT E1A sequences for the corresponding portions of HAdV-A18 and -A31, the other two species A HAdVs. Like HAdV-A12, each of these proteins contain multiple PXLXP like sequences (Figure 5A). For HAdV-A18, two of the three PXLXP like motifs conferred interaction with BS69, with the most divergent sequence (PXIXP) located at the N-terminus of CR2 failing to interact (Figure 5B). For HAdV-A31, only one of the two sequences bound, with the more C-terminal motif (PXLXS) failing to interact (Figure 5B). All constructs were expressed (Figure 5C). Thus, all members of HAdV species A E1A proteins contain one or more functional BS69 interaction SLiMs.

### 3.4. Conversion of a Non-Functional PXLXP SLiM into a Functional BS69-Binding Domain

**Figure 6:**
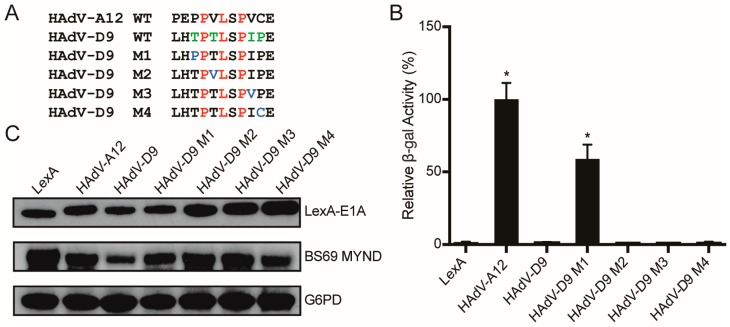
The PXLXP motif in HAdV-D9 E1A does not bind BS69, but can be functionalized by a single amino acid change. (**A**) Sequences of the WT PXLXP motif from HAdV-D9 E1A and mutants containing single amino acid changes to more closely resemble the HAdV-A12 E1A N-terminal BS69-binding motif. (**B**) Vectors expressing the motifs depicted in (A) and BS69 MYND were tested for interaction with BS69 by yeast two-hybrid analyses. The HAdV-A12 E1A N-terminal PXLXP motif was used as a positive control. Results are shown as mean ± SD of percent activity normalized to HAdV-A12 CR2 positive control, *n* = 3. Significance markers are assigned in comparison to the LexA negative control (* *p* ≤ 0.05). (**C**) Western blot analysis of expression of the fragments tested.

**Figure 7:**
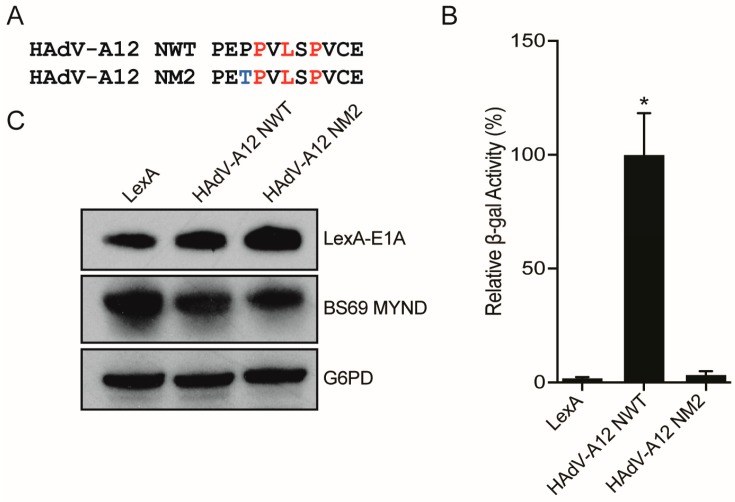
Binding by the HAdV-A12 N-terminal PXLXP motif is influenced by a proximal proline residue. (**A**) Sequences corresponding to the WT N-terminal PXLXP motif from HAdV-A12 E1A and a mutant containing a single amino acid change of proline to threonine. (**B**) Vectors expressing the motifs depicted in (A) and BS69 MYND were tested for interaction with BS69 by yeast two-hybrid analyses. Results are shown as mean ± SD of percent activity normalized to HAdV-A12 N-terminal WT positive control, *n* = 3. Significance markers are assigned in comparison to the LexA negative control (* *p* ≤ 0.05). (**C**) Western blot analysis of expression of the fragments tested.

As the non-functional PXLXP motif in HAdV-D9 E1A closely resembles the functional N-terminal BS69-binding motif present in HAdV-A12 E1A, we constructed a series of single amino acid changes in the HAdV-D9 E1A sequence to make it more closely resemble the corresponding HAdV-A12 E1A sequence (Figure 6A). This series of four mutants (M1–M4) were tested by yeast two-hybrid analysis to determine whether any of these changes were sufficient to convert this motif to a functional SLiM (Figure 6B). Interestingly, only the M1 mutant, which contains a conversion of the threonine preceding the PXLXP motif to proline interacted with BS69. All constructs were expressed (Figure 6C). Reciprocal conversion of this proline in the HAdV-A12 N-terminal BS69 SLiM to threonine abolished interaction (Figure 7A,B), despite good expression (Figure 7C). Thus, in the context of this short sequence, this preceding proline residue appears critical for binding.

### 3.5. BS69-Mediated Transcriptional Repression of E1A is Dependent on the PXLXP Motif in E1A and the MYND Domain of BS69

**Figure 8:**
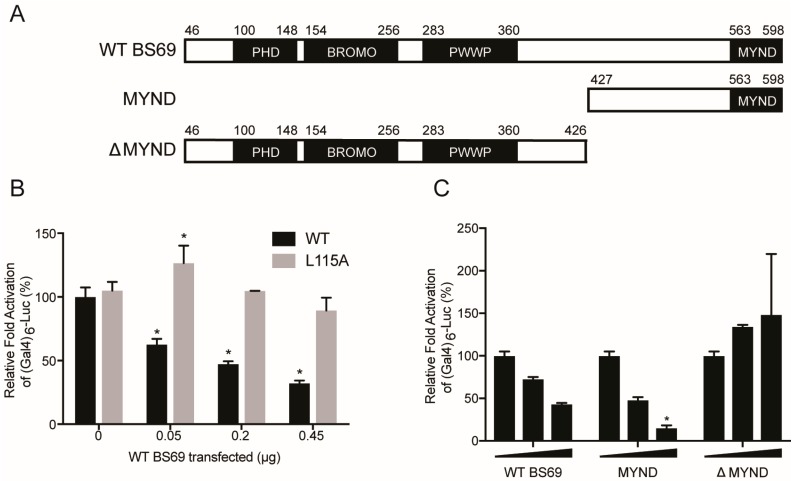
The MYND domain of BS69 inhibits E1A-mediated transcriptional activation in a dose-dependent manner dependent on the PXLXP motif. (**A**) Depiction of the domain structure of BS69 and the truncation mutants used in this study. (**B**) Luciferase assay showing repression of E1A transactivation by BS69 MYND. HT1080 cells were co-transfected with the pGL2-(GAL4)_6_-Luc reporter, either pM 13S HAdV-C5 E1A or pM 13S HAdV-C5 E1A L115A, and increasing amounts of HA BS69 WT vector. Luciferase activity was normalized by protein concentration and reported as percent activation compared to WT E1A alone. Results are shown as mean ± SD of activity normalized to WT E1A with no BS69, *n* = 2. Significance markers are assigned in comparison to the samples not transfected with a BS69 vector (* *p* ≤ 0.05). (**C**) The MYND domain of BS69 is necessary and sufficient to repress E1A transactivation. HT1080 cells were co-transfected with the pGL2-(GAL4)_6_-Luc reporter and pM 13S HAdV-C5 E1A. Cells were also co-transfected with increasing amounts (0, 0.05, and 0.25 μg) of the indicated BS69 constructs (WT BS69, MYND, or ∆MYND). Luciferase activity was normalized by protein concentration and reported as percent activation over that of an empty vector. Results are shown as mean ± SD of activity normalized to E1A with no BS69, *n* = 2. Significance markers are assigned in comparison to the samples not transfected with a BS69 vector (* *p* ≤ 0.05).

E1A is the first viral protein expressed upon HAdV infection, and one of its main functions is to activate the transcription of other early viral genes [3]. As E1A has no specific DNA-binding nor enzymatic activity, it relies on binding to host factors to facilitate its recruitment to the promoters of target genes [4,52,53]. Once tethered to a promoter, E1A is able to stimulate transcriptional initiation and elongation [54,55,56,57]. Overexpression of BS69 has been previously shown to suppress HAdV-C5 E1A-mediated transactivation, dependent on an intact PXLXP motif [22,23]. We constructed a similar luciferase assay system in mammalian cells to further study the molecular determinants of BS69 repression of E1A transactivation, and the role of the MYND domain in particular, using BS69 truncations (Figure 8A).

Consistent with a previous report, transactivation by a fusion of full-length WT HAdV-C5 E1A with the GAL4 DNA binding domain was strongly decreased by expression of exogenous BS69 (Figure 8B) [22]. This repression occurred in a dose-dependent manner. The L115 to alanine (L115A) E1A mutant, which does not bind BS69, retained transactivation that was equivalent to WT E1A. However, in contrast to WT E1A, transactivation by this mutant was not significantly reduced at any concentration of BS69 (Figure 8B). Thus, a direct interaction between the PXLXP motif in HAdV-C5 E1A CR2 and BS69 appears to be necessary for repression.

BS69 consists of four functional domains (Figure 8A). The PHD, BROMO, and PWWP domains are located in the N-terminal half of the protein, and facilitate interactions with histone tails [26,27,29]. The C-terminal MYND domain is a zinc finger that mediates protein-protein interactions, including the interaction with E1A [23,30]. To determine which domains are required for BS69-mediated repression of E1A transactivation, we tested three BS69 constructs: WT (residues 46–602), MYND (residues 427–602), and ∆MYND (residues 46–426) in the E1A repression assay. Luciferase activity decreased in a stepwise manner with increasing concentrations of the WT and MYND constructs of BS69 (Figure 8C). In contrast, transfection of the ∆MYND construct, regardless of concentration, did not significantly affect E1A transactivation of the reporter vector (Figure 8C). Thus, the MYND domain of BS69 is necessary and sufficient for repression of HAdV-C5 E1A transactivation. This suggests that the interaction between these two factors is sufficient for repression and does not require functions present in the other domains of BS69.

### 3.6. Interaction with BS69 Represses Transactivation by HAdV-C5 and HAdV-A12 E1A

The levels of transcriptional activation are dramatically different between the E1A proteins of distinct adenovirus types, yet they all interact similarly with many of the same components of the cellular RNA polymerase II transcription apparatus [58]. Thus, the cause of the differences in the relative strengths of E1A-mediated transactivation between different HAdV types is not well understood [58]. As BS69 represses E1A transactivation and binds to E1A from different species with varying affinity, we set out to determine how BS69 affected E1A-dependent transcription across different HAdV species using the GAL4 luciferase assay.

**Figure 9:**
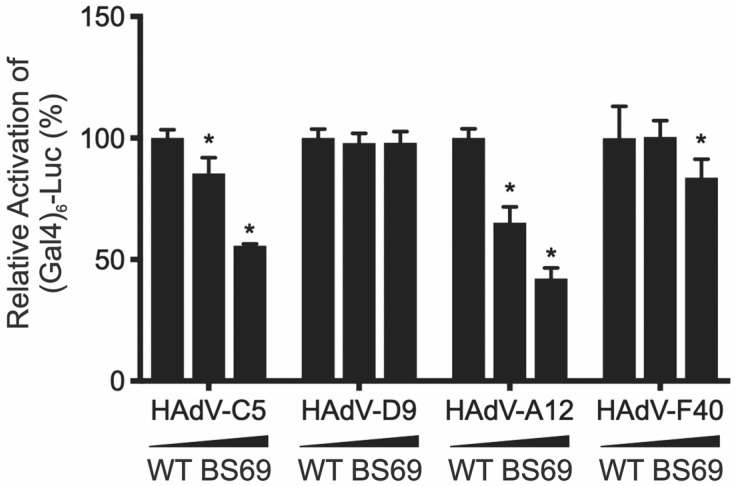
BS69-mediated repression of transactivation by HAdV-C5 and -A12 E1A. The effect of expressing BS69 on E1A-mediated transactivation was measured using luciferase assays. HT1080 cells were co-transfected with pGL2-(GAL4)_6_-Luc reporter, pM HAdV-C5, -D9, -A12, or -F40 E1A, and increasing amounts (0, 0.05, and 0.25 μg) of HA BS69 WT. Luciferase activity was normalized by protein concentration and reported as fold activation over that of an empty vector. Results are shown as mean ± SD of percent activity normalized to the E1A of each respective species with no BS69, *n* = 2. Significance markers are assigned in comparison to the samples not transfected with a BS69 vector in each respective adenovirus type (* *p* ≤ 0.05).

Like HAdV-C5 E1A, transactivation by HAdV-A12 E1A was repressed by BS69 in a dose-dependent fashion (Figure 9). In contrast, transactivation by HAdV-D9 E1A, which does not bind BS69, was not affected by BS69 at any concentration tested. HAdV-F40 E1A, which does not bind BS69, was modestly repressed by BS69 (Figure 9). However, repression was observed at the highest level of BS69 expression, which could be a secondary effect of overexpression. Taken together, the greatest effects of BS69 were observed on those E1A proteins that contain functional BS69-binding motifs.

## 4. Discussion

E1A is a small, multifunctional protein capable of directly binding to over 30 host factors with the purpose of reprogramming the cellular environment into a favorable milieu for viral replication [2,3]. E1A is also largely responsible for driving the expression of other HAdV early genes to advance the infectious cycle [59,60]. The cellular transcriptional repressor BS69 appears to be involved in both of these facets of E1A function since it binds directly to E1A and modulates E1A-mediated transactivation [22,23].

Despite the small size of E1A, it is a viral hub protein with an impressive number of connections to the cellular protein interaction network. To maximize the number of interactions using minimal coding sequence, E1A exploits numerous SLiMs, and our truncation mutation analysis has determined that the interaction between E1A and BS69 is no exception. Indeed, only eight residues are required, with the sequence MPNLVPEV spanning residues 112–118 in HAdV-C5 CR2 representing the minimal sequence for maximal interaction with BS69 (Figure 2A). The previously identified core motif PXLXP is present in this region, but without flanking residues, this sequence in isolation has no measurable ability to bind BS69 [23]. Within this sequence, P113 and L115 are crucial because mutation of either of these residues to alanine abrogated the ability of HAdV-C5 E1A to bind BS69 (Figure 3). Interestingly, substituting P117, the second proline of the highly conserved PXLXP motif with alanine did not negatively affect this interaction. Our observations agreed with results from a previous study, where residues in EBV EBNA2 analogous to E1A P113 and L115 were found to form hydrogen bonds with BS69, while the equivalent EBNA2 residue to E1A P117 only formed weaker van der Waals contacts [25]. Therefore, the P117A mutation may not be as detrimental since the replacement alanine residue may also form van der Waals contacts in a similar manner. Interestingly, HAdV-B3 E1A contains an alanine at this position, but does not bind BS69 (Figure 1A). This suggests that additional contacts mediated via adjacent residues present in HAdV-C5 E1A, but not HAdV-B3 E1A, may compensate for the replacement of this proline with alanine.

The interaction with BS69 is conserved between some, but not all the different HAdV species. Using yeast two-hybrid assays, with either the entire CR2 portion of E1A, or short peptide sequences, we found that both HAdV-C5 and -A12 E1A could interact with BS69. Interestingly, HAdV-A12 contains two functional PXLXP motifs, which are separated by 10 amino acids. When tested individually, peptides corresponding to either HAdV-A12 E1A motif was sufficient to independently bind BS69 and this required the presence of a central leucine residue (Figure 4). This is reminiscent of EBV EBNA2, which also binds BS69 and contains tandem PXLXP motifs; however, these motifs are spaced 49 residues apart in EBNA2 [25]. Inspection of the E1A sequence from HAdV-A18 and -A31, the other two species A HAdVs, reveals that like HAdV-A12 E1A, both contain two PXLXP like sequences at positions in CR2 equivalent to HAdV-A12, with HAdV-A18 containing a third motif located between them (Figure 5A). However, the most N-terminal sequence in HAdV-A18 E1A was non-functional because it has diverged to PVISP. In HAdV-A31 E1A, the C-terminal motif was non-functional due to the divergence to PQLCS. Intriguingly, all species A HAdVs have retained one or more functional copies of this SLiM.

In contrast to species A and C, the E1A proteins from species B, D, E, F, and G did not bind BS69 (Figure 1A,D). This corresponds well with the presence or absence of a PXLXP signature in the CR2 regions of these E1A proteins, with the exception of species D HAdV-D9 E1A. Although HAdV-D9 E1A contains a PXLXP signature (Figure 1C), it failed to interact with BS69 (Figure 1A,D, Figure 6, and Figure 9). Inspection of the E1A sequences of numerous other species D HAdVs revealed that the location and adjacent flanking sequence in this region is highly conserved, suggesting that the E1A proteins from none of the species D viruses bind BS69. The N-terminal flanking residue preceding the functional BS69 binding PXLXP SLiMs in HAdV-C5 E1A, HAdV-A12 E1A, and EBV EBNA2 are true aliphatic (A or P) or aliphatic-like (M). In contrast, HAdV-D9 and all other species D HAdV E1As contain a polar threonine at this position, which appears to interfere with binding since conversion of this residue to proline functionalizes this motif (Figure 6). Our previous work has shown similar differences in interactions between the E1A proteins of different HAdVs and other cellular targets, including protein kinase A, HAN11, and UBC9 [20,61,62]. These differences often result from changes in just a few key residues in their respective interaction motifs.

Repression of E1A transactivation by the BS69 MYND domain is dependent on the fidelity of the E1A PXLXP–BS69 MYND interaction. Consistent with the necessity for a direct interaction, the L115A HAdV-C5 E1A mutant was unable to bind BS69 and was resistant to BS69-mediated inhibition (Figure 8B). In our analysis, the N-terminal PHD, BROMO, and PWWP domains of BS69 were not required to repress transactivation by E1A, which required only the MYND domain (Figure 8C). This observation is consistent with previous reports regarding EBNA2 [25]. Indeed, the ability of BS69 to repress transactivation is not just restricted to E1A because EBNA2 and ETS2 are similarly affected [25,36]. We also show here that BS69 can repress transactivation by the E1A proteins from other species, as long as they contain a functional PXLXP motif that confers binding to BS69 (Figure 9).

## 5. Conclusion

In summary, our data shows that the E1A proteins from HAdV-C5 and -A12 bind the cellular repressor protein BS69. This occurs via a SLiM containing a conserved PXLXP motif, which is present in CR2 of E1A. This SLiM confers interaction with the MYND domain of BS69. Functionally, the interaction with BS69 represses transactivation by E1A, and this may serve as a means to regulate viral gene expression during infection. Furthermore, comparison of the E1A sequences from multiple HAdV types reveals that at least portions of the disordered regions of E1A are rapidly changing, with some types containing more than one PXLXP motif and many others with near matches that are non-functional. Importantly, as little as one single amino acid substitution was sufficient to convert a non-functional SLiM in HAdV-D9 E1A into a functional motif, elegantly illustrating how rapidly SLiMs can evolve. As such, these studies of E1A provide support for the proposed process of *ex nihilo* SLiM evolution [63], which is the evolution of novel functional short protein interaction motifs from “nothing” based on random mutation and selection in structurally disordered protein regions.
